# Interprofessional education in healthcare professions – implementation status and preferences from the perspective of teaching staff

**DOI:** 10.3205/zma001814

**Published:** 2026-02-17

**Authors:** Jann Niklas Vogel, Annemarie Bagner, Matthias Müller

**Affiliations:** 1 Neubrandenburg University of Applied Sciences, Department of Social Work, Education and Childcare, Neubrandenburg, Germany

**Keywords:** interprofessional learning, interprofessional education, interprofessionalism, healthcare training, interprofessional collaboration, skills

## Abstract

**Background:**

Interprofessional thinking and behaviour are the key to high-quality and efficient healthcare provision. This requires specific skills which can be developed through interprofessional teaching and learning. This article sets out methods of implementation and preferences of interprofessional teaching in the education of healthcare professions from the perspective of teaching staff.

**Methodology:**

Semi-structured interviews (n=11) were carried out with teaching staff from four public schools for healthcare professions in Mecklenburg-Vorpommern. The data was evaluated using the methodological approach of content-structuring qualitative content analysis.

**Results:**

The implementation of interprofessional teaching and learning varies depending on the institution and teacher. It takes place in classrooms, in nursing skills labs, on excursions and project days, but requires a high degree of planning. Teaching staff favour case scenarios with a clear structure, which can be worked on by several professional groups or from various perspectives. Reflecting on profession-specific perspectives plays a particularly important role here. Digital formats such as blended learning, simulation labs as well as virtual and augmented reality are cited as supplementary learning formats, but often fail in their implementation due to a lack of technical equipment.

**Discussion & conclusion:**

In addition to political and institution-specific contexts for interprofessional teaching and learning, interprofessional educational formats are also required. These should be based on case scenarios and be modular so that individual teaching and learning content can be applied to various contexts. The integration of OER as well as digital learning formats can support both the implementation and preparation and follow-up of interprofessional learning activities.

## Zusammenfassung

**Hintergrund:**

Berufsgruppenübergreifendes Denken und Handeln gelten als Schlüssel für eine qualitativ hochwertige und effiziente Gesundheitsversorgung. Dies erfordert spezifische Kompetenzen, welche durch interprofessionelles Lehren und Lernen angebahnt werden können. Der vorliegende Beitrag zeigt Umsetzungswege und Präferenzen interprofessioneller Lehre in der Ausbildung von Gesundheitsberufen aus Lehrendenperspektive.

**Methodik:**

Es wurden Leitfadeninterviews (n=11) mit Lehrenden aus vier öffentlichen Schulen für Gesundheitsfachberufe in Mecklenburg-Vorpommern durchgeführt. Die Datenauswertung erfolgte über den methodischen Ansatz der inhaltlich strukturierenden qualitativen Inhaltsanalyse.

**Ergebnisse:**

Die Umsetzung interprofessionellen Lehrens und Lernens variiert je nach Einrichtung und Lehrperson. Es findet im Klassenunterricht, Pflegekabinett, bei Exkursionen und Projekttagen statt, erfordert jedoch hohe Planungsaufwände. Lehrkräfte bevorzugen dabei Fallszenarien mit klarer Struktur, die von mehreren Berufsgruppen oder perspektivübergreifend bearbeitet werden können. Dabei nimmt die Reflexion berufsspezifischer Perspektiven einen besonderen Stellenwert ein. Digitale Formate wie Blended Learning, Simulationslabore sowie Virtual- und Augmented Reality werden als ergänzende Lernformate genannt, scheitern in der Umsetzung jedoch häufig an fehlender technischer Ausstattung.

**Diskussion & Schlussfolgerung:**

Neben politischen und einrichtungsspezifischen Rahmenbedingungen für interprofessionelles Lehren und Lernen bedarf es berufsgruppenübergreifender Bildungsformate. Diese sollten auf Fallszenarien basieren und modular gestaltet sein, sodass einzelne Lehr- und Lerninhalte auf unterschiedliche Rahmenbedingungen übertragen werden können. Die Integration von OER sowie digitalen Lernformaten kann dabei sowohl die Durchführung als auch die Vor- und Nachbereitung interprofessioneller Lernangebote unterstützen.

## 1. Background

The German healthcare system is faced with the challenge of maintaining a high level of healthcare despite the increasing complexity of care delivery [1]. One key approach to solving this involves interprofessional collaboration between healthcare professionals in order to pool professional skills and plug gaps in care. The WHO [2] defines interprofessionalism as teaching and work that occurs when at least two professions work together and learn from each other. Interprofessional teaching and learning (IPTL) has become an established educational concept intended to promote specific skills during education for future interprofessional collaboration [3], [4]. IPTL goes beyond parallel learning and requires structured learning situations which enable the exchange of ideas and mutual reflection on profession-specific perspectives. It is not strictly necessary for IPTL to be carried out by teaching staff from different professions. The key factor is that the learning activity is aimed at at least two professional groups, with interprofessional exchange at its core. A previous literature view [5] demonstrated that IPTL is implemented in very different ways in practice, both in terms of duration (from 120 minutes to several contact hours per week per semester) and methods used. Case-based learning, simulations and small group work are often used [5]. At the same time, it has been shown that IPTL often remains confined to lower levels of interaction such as exchange-based learning, and more complex forms such as action-oriented or observation-orientated learning are less commonly implemented [5]. Most often, only two professional groups are involved, primarily nursing and medicine [5]. Incorporation in the curriculum and alignment with didactic concepts such as backward design are considered key success factors which are, however, only rarely implemented [5].

With this in mind, the question to be asked is how IPTL is implemented in professional educational practice, what framework conditions this requires and how teaching staff assess its potential and challenges. A sub-project of the joint project Campus BWP MV, the University of Rostock and the Neubrandenburg University of Applied Sciences addressed these questions. This study focuses on the following questions:

1. How is IPTL implemented for healthcare professions at vocational schools?
2. What are the preferences and needs of teaching staff in terms of IPTL?

The findings are intended to help develop and apply needs-based and practical scenarios for IPTL in vocational education. 

## 2. Method

Interviews were conducted in the form of expert interviews [6]. Data was collected from teaching staff at vocational schools in Mecklenburg-Vorpommern (MV) which train healthcare professionals. This group of people were considered experts as they have special knowledge of healthcare didactics. Expert interviews usually take place in a semi-structured format [6]. An interview guide was therefore developed based on a previous literature review [5] and designed in a focus group including the authors JV, AB and MM as well as a research assistant. The interview guide developed consists of five topics (see table 1 (Tab. 1)).

### 2.1. Interview guide topics

The open-ended questions in the interview guide ensured that interviewees had adequate scope to respond within their own framework of relevance. Data collection was approved by the Ministry of Education and Daycare Facilities for Children of Mecklenburg-Vorpommern on the condition that the interviews were not recorded, but rather logged. The interviews took place from June 2022 to April 2023. A period of 30 minutes was factored in for each interview. During the interview, the interviewer immediately noted key statements and also documented, to a small extent, verbatim statements (simultaneous protocol). Questions were asked to clarify anything unclear. In this way, the statements were communicatively validated during the interview itself [7]. The interview notes were promptly processed, producing a complete and well-structured record. 

### 2.2. Data evaluation

Data was prepared and evaluated anonymously using a category-based approach. The methodological approach of content-structuring qualitative content analysis [8] was used to prepare and evaluate the data material. The methodology enables a deductive-inductive approach, allowing research findings from the previous literature review [5] to be incorporated. The methodology is in line with the exploratory objective of the study, which does not involve assessing the research data or reducing it to typologies [8], [9]. All steps of data evaluation were electronically assisted via the software MAXQDA (2022 version). In the first step, deductive categories were created using the interview guide. The second step involved creating categories based on the data material. After differentiation of the category system, the interview data was completely coded [8]. The evaluation was carried out by two researchers, who collectively compared the coding and category system to ensure validity. The coded segments provided an overview that linked the codes from the interview transcripts to the individual main and subcategories [8]. A description together with an illustrative example was formulated for all the codes in order to be able to better review, organise and interpret the data material. The results from the data analysis were then prepared using a category-based approach.

## 3. Results

A total of four vocational schools in the healthcare sector in MV took part in the interviews. Interviews took place on-site/in-person (n=9) or using a video conference tool (n=2) and lasted an average of 25 minutes. All interviews conducted were incorporated in the data analysis. The sample (n=11) consisted predominantly of female participants (n=10). As there was no indication of different gender-specific statements in the interviews, the dominance of female respondents in the sample was accepted and no further questioning carried out. Seven main categories with a total of 12 subcategories were drawn up as part of the data evaluation.

### 3.1. Current implementation of IPTL

The teaching staff explained that they had already been practising IPTL in healthcare education for some time: *“We’ve been using IPTL for a while. For around 10 years” (pg. 3, line 2).* In this context, the teaching staff also teach courses in other vocational training professions (e.g. physiotherapists for nursing specialists (PFF)). Teaching sometimes takes the form of team teaching where one of the two staff members assumes the main teaching responsibilities and the other staff member provides support as needed. Alongside special teaching methods, there is collaboration with external teachers (such as practical instructors), institutions (such as the medical supply store) or other external service providers (such as de-escalation instructors). In general, IPTL is suitable for vocational schools in the healthcare sector whenever there are shared curricular content and interprofessional learning opportunities: *“CE6: Taking action in emergencies (connection: nursing specialists (PFF) & paramedics)” (pg. 1, line 50).* Depending on the type and scope of IPTL, these take place either during regular teaching hours or on special project days. 

### 3.2. Problems and risks in implementing IPTL

From the perspective of the teaching staff, the core curricula limit the opportunities for interprofessional teaching: *“The core curriculum in nursing hardly leaves any space or time for IPTL” (pg. 1, line 32).* IPTL is not incorporated in the curriculum. Time coordination, staff shortages or the school’s own curriculum often constitute obstacles in implementing IPTL. Teaching staff describe having a “bad feeling” when colleagues from another profession cover a specific teaching topic for IPTL and no reciprocal contribution can be made. Moreover, interprofessional collaboration with some healthcare professions (such as between nursing specialists and medical assistants) is considered minimal and thus of limited relevance. During the coronavirus pandemic, protective measures meant that many forms of implementing IPTL, such as collaboration with external service providers (such as the hospice, medical supply store, or pathology) were not feasible. 

### 3.3. Objectives of IPTL

From the teaching staff’s perspective, IPTL has various aims. One key aspect is *“getting to know the other professional groups, their duties and fields of application” (pg. 5, line 18).* Interprofessional knowledge is intended to break down prejudices, expand one’s own profession-specific view and foster mutual support and successful collaboration in professional practice. One important aspect is reflecting on interprofessionalism in order to ensure that professional collaboration is purposeful and effective. 

### 3.4. Consequences and advantages of IPTL

In the view of the interviewees, IPTL brings about an exchange of ideas between professional groups, which provides a holistic view of healthcare situations. IPTL should make students aware *“[...] that you work together with different professional groups (such as with sports therapists)” (pg. 5, line 22).* Cross-occupational understanding lays the foundations for successful collaboration and can form the basis for subsequent mutual support in professional practice. This can help create a better working environment and increase job satisfaction. Several teaching staff indicated in the interview that IPTL can result in *“better healthcare provision” (pg. 1, line 23).* Teaching staff also expand their own teaching skills and expertise as a result of IPTL. 

### 3.5. Design preferences for IPTL

IPTL should be designed in the form of case scenarios in the view of the respondents and have a clear structure (preparation, implementation, follow-up). Reference to the curricular units (CU) of nursing education provides a structure by topic and a chronological order. As part of case-based learning, teaching staff should initially introduce students to the topic so that they can work on the case in an interprofessional manner or with an interprofessional perspective. IPTL is intended to appeal to various senses of the students in order to make the delivery of the learning content more engaging, make learning easier and increase student outcomes. The transfer of profession-specific perspectives is especially important and can be promoted via the presentation of group results or discussion and reflection sessions. In terms of grading, teaching staff said that no grades should be awarded as part of IPTL as this would inhibit the openness of the teaching and learning process. In addition to applying IPTL in the classroom, IPTL is also applied in practice in the nursing skills lab, a dedicated training room for practising nursing procedures in an educational setting: *“There are three mannequins in the nursing skills lab, where the professional groups work together and, for example, nursing procedures are demonstrated” (pg. 3, line 36).* The nursing skills lab provides a safe learning environment for interprofessional healthcare that encourages learning from mistakes. Looking ahead, the interviewees expressed a desire to develop IPTL for new learning concepts, such as learning in the simulation laboratory (SimLab). Furthermore, respondents expressed interest in implementing IPTL using Augmented Reality (AR) and Virtual Reality (VR), although some of the vocational schools admitted not having adequate technical equipment for this. The newly developed scenarios for IPTL are to be initially tested and then further developed. The time frame for IPTL is determined by the relevant content. For example, teaching staff indicate a time frame of around 10 minutes for IPTL in the SimLab. Depending on the number of professional groups involved, the complexity of the case and the task(s), IPTL can last between 45 minutes and a week. The teaching and learning sequences should be kept relatively short and include reflective parts in the form of repetitions. For extensive content, teaching staff recommend scheduling IPTL in a project week: *“IPTL is best carried out in a project week as no other content is taught during this period.” (pg. 2, line 41). *

### 3.6. Professional groups for IPTL

Various professional fields were represented at the vocational schools surveyed in the healthcare sector, with IPTL being designed differently depending on the institution. Interprofessional educational cooperation took place between physiotherapy and nursing specialists, occupational therapy and nursing specialists, nursing specialists and cooks, and physiotherapists and radiology technicians (MTR).

### 3.7. Other

For the practice-oriented design of IPTL, teaching staff require targeted initial, continuing, and advanced training programmes. Some interviewees noted that teaching staff might have little interest in corresponding formats: *“Advanced training is required; the target group probably only has limited interest” (pg. 10, line 32).* In the view of one interviewee, a special working group for IPTL would have limited appeal and be unlikely to succeed alongside already existing working groups. Some vocational schools in the healthcare sector therefore specifically deploy trainee teachers to develop new teaching and learning formats: *“The advantage of our teacher training school is that trainee teachers are involved in developing the curricular units” (pg. 11, line 18).*

## 4. Discussion

As shown in the previous literature review [5], the use of IPTL depends on the appropriate time, the incorporation into teaching and the intended objective in training [10]. The present results supplement these findings through insights into implementation forms and preferences of teaching staff at vocational schools in the healthcare sector in MV. IPTL takes place there both in regular teaching and in specific learning settings (such as in the nursing skills lab or on excursions). These examples illustrate the application-oriented use of IPTL with limited resources. It is clear that occasional learning opportunities are not enough to promote interprofessional skills in the long term [10]. Teaching staff emphasise that IPTL must be integrated into the curriculum and implemented over extended periods, as also advocated by Sottas et al. [10]. A key result of the study is the preferred use of case-oriented learning settings. This method is deemed particularly suitable for making interprofessional learning practical and comprehensible. This is also confirmed by the research which describes such scenarios as effective for developing skills [11]. The results also indicate that there is a growing interest amongst respondents to make greater use of new learning formats (SimLab, AR, VR) for IPTL. However, this often fails as a result of inadequate technical equipment. The study makes clear that IPTL not only has a positive impact on students but also supports teachers themselves in their professional development. In addition to expanding teaching skills, IPTL helps teachers reflect on and further develop subject-related content. As Sottas et al. emphasise, interprofessional collaboration is not only a methodological challenge, it also requires a collective understanding of roles and a conscious engagement with communication patterns and power relations between the professional groups [10].

This study was based on the core academic criteria for qualitative research according to Steinke [12], [13]. To preserve intersubjective transparency, the data evaluation was documented using MAXQDA and the content analysis carried out by two researchers using a codified method. Empirical grounding [13] is ensured by the codified method, reference to the previous literature review, the deductive-inductive approach as well as the derivation of findings. Despite the aforementioned quality criteria, the study has limitations that relate to the sample composition, the creation of the interview guide, the type of data collection and evaluation as well as the authenticity of responses. As participation in the interviews was voluntary, there is potential for distortion as primarily committed and motivated people took part who tend to have more active roles within their institutions. It cannot be ruled out that interviews with teaching staff at other vocational schools would have led to additional findings or other priorities. The composition of the sample therefore limits the transferability of the results to other sites of education in Germany. Another limitation involves the written record of the interviews. This method was appropriate for the research project as the focus was on substantive content rather than on interpretive understanding [7]. All interviews were conducted and documented by a person with experience in empirical interview research, which meant that distortions due to inconsistent implementation could be reduced. At the same time, it cannot be ruled out that the one-sided perspective in documentation has resulted in omissions in content or the loss of nuance in responses.

## 5. Conclusion

The results illustrate that it is a challenge in vocational training to implement a harmonised approach to IPTL. Instead, flexible formats are required, which can be adapted to the respective staffing and organisational conditions as well as the curriculum. Teaching staff favour practical, case-oriented approaches that build on existing teaching structures and enable dialogue between professional groups. In this context, the joint project Campus BWP MV has worked on developing modular, practical and adaptable teaching scenarios intended to support the flexible integration of interprofessional content in teaching practice. A key element involves open educational resources (OER), which can be used for implementation as well as preparation and follow-up. OERs can facilitate the long-term and effective implementation of IPTL because IPTL can be exchanged between educational institutions and professional practice and further developed. Some teaching staff’s desire for structural support within schools, such as working groups or internal coordination within schools, in order to be able to continuously further develop interprofessional learning opportunities is consistent with Nock [14] and is recommended. Here, incorporating IPTL in the curriculum, for example based on the framework according to Sottas et al. [10], can help gradually develop skills and secure them in the long term. The interviews suggest that teaching skills and subject-matter coordination should be given greater consideration in interprofessional collaboration in teacher training. Further research is required to evaluate design aspects and the impact of IPTL as well as its effects on collaborative professional practice.

## Abbreviations

- CU = curricular units
- IPTL = Interprofessional Teaching and Learning
- MTR = radiology technician
- MV = Mecklenburg-Vorpommern
- OER = Open Educational Resources
- PFF = nursing specialist
- SimLab = simulation laboratory

## Acknowledgements

We would like to extend our sincere thanks to all participating vocational schools and the teaching staff for volunteering to be interviewed.

## Funding

This work was part of the joint project “CAMPUS BWP MV”. The project was funded by the Federal Ministry of Education and Research as part of the joint “Teacher Training Quality Campaign” of the federal and state governments. Funding code: 01JA2023A

## Authors’ ORCIDs

- Jann Niklas Vogel: [0000-0002-8937-6172]
- Matthias Müller: [0000-0001-5694-0083]

## Competing interests

The authors declare that they have no competing interests. 

## Figures and Tables

**Table 1:**
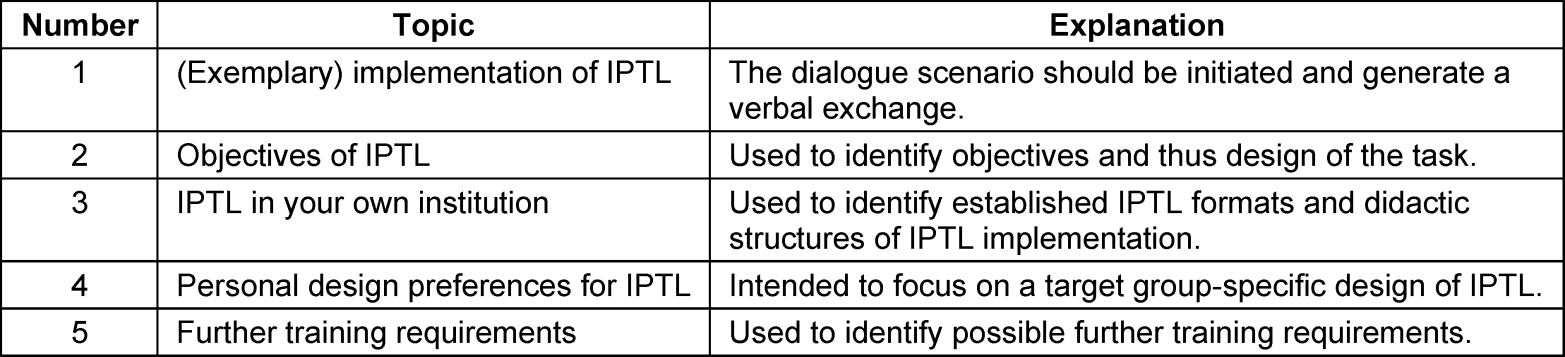
Interview guide topics
